# Ventilation

**Published:** 1882-07

**Authors:** 


### VENTILATION.

**Don't** let the reader be astounded out of his propriety and declare us insane, because we tell him, it is more important to sleep with a window up in mid winter, than in summer-time. How few people have the gift of thinking! how many have the gift of gab, in the inverse ratio! The less a man thinks, the more he can talk; that is the very reason why our household divinities can discourse indefinitely, ad infinitum, and the other side of it. Whoever heard of a man taking cold who slept in *all out doors?* Well, if sleeping in all out doors, does not give a man a cold, how can sleeping in a part of all out doors give him a cold? Is not that conclusive? Surely none of the unthinking multitude could ask for a more convincing argument than this. But as only the thinking few take a journal like this, we will give a mere hint of an argument, with the carrying out of which, they may amuse themselves in a leisure hour. Pure carbonic acid gas is deadly, it kills in five minutes. In sleeping we breathe out this gas, and a close room confines it, warmth makes it rise to the ceiling, cold condenses and keeps it near the floor. *Verb. sat.*

---

### HOW TO ARGUE.

It is *prima facie* evidence of a low intelligence or a low nature, or both, in the man who resorts to personalities in controversy; and if I retaliate in kind, I but sink myself to his level, to rise from it no more.

Conscious power is calm. An admirable illustration of this was given by the late Mr. Henderson, a Scotchman, and an eminent Oxford scholar. A collegian, proud of his logical abilities, was anxious to meet Mr. Henderson, but shortly losing his self-possession, he dashed a glass of wine in the Scotchman's face. The latter, coolly wiping it away, replied: "*Sir, this is a digression, now for the argument!!*"

There is perhaps the shadow of an apology for letting drop an epithet or a sarcasm in an oral dispute, but when persons use these, with all the advantage of writing out a controversy, when they become deliberately personal, in the quiet of their own library, we instinctively pity them, as inheriting a rude nature which time and opportunity are powerless to polish.

